# Social participation and change in walking time among older adults: a 3-year longitudinal study from the JAGES

**DOI:** 10.1186/s12877-022-02874-2

**Published:** 2022-03-22

**Authors:** Shiichi Ihara, Kazushige Ide, Satoru Kanamori, Taishi Tsuji, Katsunori Kondo, Gemmei Iizuka

**Affiliations:** 1 Medical School, Chiba University, 1-8-1, Inohana, Chuo-ku, Chiba-shi, Chiba, 260-0856 Japan; 2 Center for Preventive Medical Sciences, Chiba University, Inage-ku, Chiba, Japan; 3 Teikyo University Graduate School of Public Health, Tokyo, Japan; 4 Department of Preventive Medicine and Public Health, Tokyo Medical University, Tokyo, Japan; 5 Faculty of Health and Sport Sciences, University of Tsukuba, Bunkyo City, Tokyo, Japan; 6 Center for Gerontology and Social Science, National Center for Geriatrics and Gerontology, Obu, Aichi Japan; 7 Graduate School of Medical and Pharmaceutical Sciences, Chiba University, Chiba, Japan

**Keywords:** Physical activity, Volunteering, Sports, Hobby, Paid work, Healthy aging, Social capital, Social network, Japan

## Abstract

**Background:**

Among all physical activities, walking is one of the easiest and most economical activities for older adults’ mental and physical health. Although promoting social participation may extend the walking time of older adults, the longitudinal relationship is not well understood. Thus, this study elucidates the relationship between nine types of social participation and change in walking time during a 3-year follow-up of older adults.

**Methods:**

We conducted a 3-year community-based longitudinal study of independent older adults in Japan. From the 2016 and 2019 surveys, we extracted 57,042 individuals. We performed multiple regression analyses, estimating associations between change in walking time after three years and nine types of social participation in 2016: volunteer, sports, hobby, senior, neighborhood, learning, health, skills, and paid work. We conducted subgroup analysis stratified by walking time in 2016 (i.e., < 60 or ≥ 60 min/day).

**Results:**

The mean (standard deviation) change in walking time for 3 years was − 4.04 (29.4) min/day. After adjusting potential confounders, the significant predictors of increasing or maintaining walking time (min/day) were participation in paid work (+ 3.02) in the < 60 min/day subgroup; and volunteer (+ 2.15), sports (+ 2.89), hobby (+ 1.71), senior (+ 1.27), neighborhood (+ 1.70), learning (+ 1.65), health (+ 1.74), and skills (+ 1.95) in the ≥ 60 min/day subgroup compared with non-participants.

**Conclusions:**

Paid work and community activities may be effective for maintaining or increasing walking time among older adults with less (< 60 min/day) and sufficient (≥ 60 min/day) walking time, respectively.

**Supplementary Information:**

The online version contains supplementary material available at 10.1186/s12877-022-02874-2.

## Background

Improving exercise habits worldwide has become an essential aim in creating a healthy society with a long lifespan [1–3]. In 2016, the World Health Organization reported that more than 1.4 billion adults worldwide were underexercized [4]. Notably, increasing physical activity in older adults can improve physical and mental health [4–6]. Among physical activities, walking is one of the easiest and most economical activities for older adults to conduct daily exercise, [7, 8] which also provides other physical and mental health-promoting effects [6, 9, 10].

From the viewpoint of maintaining and improving health, the Japanese Ministry of Health, Labor and Welfare, and the literature have indicated that it is desirable for older individuals to walk an average of 6,000 to 8,000 steps per day [11–13]. In studies on walking in older adults, 1 min of walking resulted in approximately 100 steps; [12] thus, Japanese older adults should walk approximately 60 min per day. However, it failed to increase the amount of walking recommended by educational campaigns, such as Health Japan 21 (2000–2010), conducted to improve individuals’ health, including an increase in the amount of physical activity [14]. Hence, identifying factors that increase and maintain the walking time of older adults such that their average daily walking time is at least 1 h is essential. Especially in the United Kingdom, local governments and physicians have focused on the positive impact of social participation on health behavior and health and provided patients opportunities for social participation as one option for treatment and improving their health, which is called “social prescribing” [15, 16]. A longitudinal study investigating the relationship between social participation and physical activity demonstrated that individuals who participate in social engagement regularly are more likely to maintain or increase physical activity than those without social participation, [17] suggesting that promoting social participation can be an effective intervention for healthy behavior. In this context, the Japanese government formulated a strategy for encouraging community-dwelling older adults to participate in local social activities as part of a health promotion campaign. However, what remains unclear is which types of social participation, such as volunteering, sports, hobby groups, or neighborhood associations, have a positive effect on maintaining or improving walking time. From this viewpoint, identifying the specific types of social participation that contribute to promoting and maintaining walking time for older adults is vital. Additionally, most studies on social factors that affect physical activity have been cross-sectional, and few have been large-scale longitudinal studies [18, 19].

The purpose of this study was to examine the relationship between nine types of social participation and change in walking time during a 3-year follow-up among older adults, stratifying the average daily walking time by less than or equal to 60 min or more than 60 min.

## Materials and methods

### Sample

**Fig. 1:**
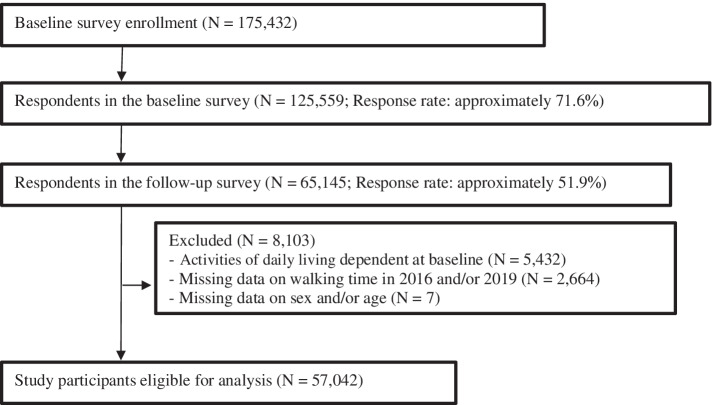
Flowchart of participants

We obtained the data from the Japan Gerontological Evaluation Study (JAGES), which repeated every 3-year community-based survey and followed up older adults in Japan [20, 21]. JAGES has collected baseline data from functionally independent older adults aged 65 years and over who were ineligible for public long-term care insurance benefits [20]. We used the longitudinal panel data from two surveys conducted in 2016 and 2019 in 23 municipalities. In both surveys, a self-administered questionnaire was mailed to each participant. Through these two surveys, we obtained valid responses from 65,145 respondents, excluding those who were either deceased, had relocated, or had a certification of eligibility for benefits of long-term care insurance. Of the 65,145 respondents, 5,432 were excluded because of activities of daily living (ADL) limitations at baseline. Furthermore, we excluded 2,671 individuals because their information on their sex and daily walking time in 2016 and/or 2019 was missing. After all exclusions, the final sample for analysis was 57,042 respondents (Fig. 1).

### Measurements

#### Walking time

As a dependent variable, we evaluated the differences in walking time from 2016 to 2019. In both surveys, participants were asked about their mean daily walking time (< 30, 30–59, 60–89, or 90 min/day or more). We converted these categories into continuous variables:15, 45, 75, and 105 min per day [22]. As the dependent variable, we used the difference in walking time from 2016 to 2019.

#### Social participation

To survey the daily life and functional health of older adults (those more than 65 years old), the Japanese local government conducted “a survey of needs in the spheres of daily life,” and several similar surveys have been performed by many institutions, including the JAGES. These surveys inquired on the situation of the social participation of older adults, which was categorized in detail (e.g., volunteering group, hobby group, and senior citizen clubs). Many studies were also conducted on identifying the positive factors of healthy aging. To obtain independent variables, we evaluated the status of social participation in 2016. In the survey, we asked about nine types of social participation: volunteer groups (volunteer), sports groups or clubs (sports), hobby groups (hobby), senior citizen clubs (senior), neighborhood associations (neighborhood), learning or cultural groups (learning), long-term care prevention or health-promoting activity groups (health), activities to teach skills or pass on experiences to others (skills), and paid work. Study participants chose for six categories their frequency of participation (four or more times per week, two or three times per week, once per week, once to three times per week, a few times per year, and never) for each type of organization. We created three categories for each type of social participation: participation (participate more than a few times a year), no participation (never), and missing (invalid or missing response). Organizations especially characteristic of Japan among the aforementioned types were senior citizen clubs. Japanese senior citizen clubs conduct wide-ranging activities, for example, group activities such as sports, hobbies, cultural activities, and performing arts [23]. Senior club activities differ from other activities because it is a nationwide association organized by the Japan Federation of Senior Citizens Clubs, whereas other activities are organized by local governments or nonprofit organizations. These clubs have conducted their activities in cooperation with local government welfare departments and/or similar public agencies. Some clubs organize group walking activities for local senior residents as a part of their daily activities [24]. The nine types of social participation were selected on the basis of the types used in “the survey of needs in the spheres of daily life,” which was conducted by the Japanese local government as a survey on the daily life and functional health of older adults (more than 65 years old) [25] and the types used in the Japan General Social Survey [26].

#### Confounders

We evaluated potential confounding factors considered to be correlated to social participation and walking time. These factors were demographic variables, socioeconomic status, and health behavior. In the 2016 survey, these factors were included in the analyses as covariates. The demographic variables and socioeconomic status indicators were sex, age (65–69, 70–74, 75–79, 80–84, or ≥ 85 years), marital status (married, widowed, divorced, never married, or others/missing), educational attainment (< 10, 10–12, ≥ 13 years, or others/missing), household income (< JPY 2,000,000, 2,000,000–3,999,999, ≥ 4,000,000, or missing; JPY 1 million is equivalent to USD 10,000). Health behavioral information comprised body mass index (< 18.5, 18.5–24.9, ≥ 25.0 kg/m^2^, or missing), tobacco-smoking habits, and alcohol consumption. We also evaluated instrumental ADL (IADL) by using the Tokyo Metropolitan Institute of Gerontology Index of Competence (TMIG-IC) to measure the living ability of older adults residing in the area [27]. In this study, to measure IADL, we used the five-item TMIG-IC, which is based on the Lawton IADL scale. In this scale, five activities that individuals may perform in daily life were evaluated: using public transportation, shopping for daily necessities, preparing meals, paying bills, and managing deposits at a bank or post office. Each item was scored 1 for *yes* (able to do) or 0 for *no* (unable to do). Participants whose total score was less than 4 were defined as *dependent*, and those with a total score of 5 were defined as *independent*.

### Statistical analysis

We performed multiple regression analyses to examine associations between the nine types of social participation and the difference in walking time from 2016 to 2019. The following two models were constructed based on each social participation type. Each status of the nine types of social participation in 2016 was included in the crude model. Moreover, the nine types of social participation were introduced in each model separately. In Model 1, we added sex and age to each crude model as demographic factors. In Model 2, all covariates were added to Model 1 to adjust the IADL, socioeconomic status, and health-related behaviors, which we considered confounders on walking time. To determine factors that improve walking time, especially among older individuals whose walking time is low, we separated the sample into two groups: the < 60 group (walks < 60 min/day at baseline survey) and the ≥ 60 group (walks ≥ 60 min/day). We used a walking time of 60 min per day as a cut-off criterion given that the literature recommends walking for more than 6,000 steps per day, [11–13] which can be calculated to approximately 70 min per day. Moreover, given the fact that the average walking steps of Japanese older adults is 4,600–5,400 per day, [13] which is equivalent to approximately 50–60 min, the proposed cut-off criterion can also be interpreted as those who walk less and more than the average.

All variables were set as dummy variables. Based on the literature, [28] a “missing” category was used in the analysis to account for missing values in response to questions. This method, called the indicator method, is widely used by epidemiologists for managing missing data [29]. The indicator method also has higher precision than complete-case analysis because a record is included for every individual in the dataset. We also conducted sensitivity analyses defining social participation as attendance once a month or more often in the specific group’s activity.

We used RStudio Version 1.2.5042. (Mozilla/5.0 Macintosh; Intel Mac OS X 10_16_0), and *p* value < 0.05 was considered to be statistically significant for all statistical analyses.

## Results

The number of participants enrolled in our study was 57,042 (34,542 in the < 60-year group and 22,500 in the ≥ 60 group). The mean (standard deviation) change in walking time for 3 years was − 4.04 (29.4) min/day for the total study participants, 6.24 (24.36) min/day in the < 60 group and − 19.83 (29.48) min/day in the ≥ 60 group. Table 1 and the supplemental material 1 present the respondents’ characteristics at baseline. Changes in walking time from 2016 to 2019 were calculated in the < 60 group (mean: + 6.24 min/day, SD: 24.36) and the ≥ 60 group (mean: − 19.83, SD: 29.48). In each regression model, we examined the adjusted generalized VIF to rule out the possibility of collinearity. All numbers of generalized VIF were < 2.00 for models 1 and 2.

Results of regression models based on the complete dataset are shown in Tables 2 and 3. In the analysis of the crude model and Models 1 and 2, paid work (work) was the only social participation that had a positive difference in the amount of change in walking time after 3 years in the < 60 group. In the ≥ 60 group, for all types of social participation except paid work, we observed a positive difference in the amount of change in walking time after 3 years. In Model 2, significant predictors of the positive difference in the amount of change in walking time after 3 years were participation in paid work (work:β = 3.02, 95%CI 2.41–3.63) in the < 60 group, and volunteer (β = 2.15, 95%CI 1.23–3.06), sports (β = 2.89, 95%CI 2.04–3.73), hobby (β = 1.71, 95%CI 0.89–2.54), senior (β = 1.27 95%CI 0.14–2.40), neighborhood (β = 1.70, 95%CI 0.87–2.52), learning (β = 1.65, 95%CI 0.58–2.72), health (β = 1.74, 95%CI 0.65–2.82), skills (β = 1.95, 95%CI 0.85–3.05) in the ≥ 60 group.

## Discussion

Among the nine types of social participation (more than a few times a year), the significant predictors of increasing or maintaining daily walking time (min/day) were participation in paid work in the < 60 min/day subgroup and all types of social participation except for paid work (volunteer groups, sports groups or clubs, hobby groups, senior citizen clubs, neighborhood associations, learning or cultural groups, long-term care prevention or health-promoting activity groups, activities to teach skills or pass on experiences to others) in the ≥ 60 min/day subgroup compared with non-participants at baseline.

Earlier reports indicated that social participation had a protective association with physical activity, [17, 30] and our finding is consistent with that finding. Besides, in our study, we examined each type of social participation separately. As sensitivity analyses, we also conducted analyses defining social participation as attendance more than once a month in the specific group’s activity with similar tendency being seen. All social participation other than paid work showed a positively significant result among respondents who walked 60 min or more in 2016. On the basis of this finding, several mechanisms can be considered. First, social participation promotes social networks and social support. Older adults who participate in social activities can benefit from social support and network, such as making friends who go outside and walk together and being positively influenced to increase or maintain their physical activity [17, 28, 30, 31]. Second, social participation leads to various physical and mental benefits, e.g., preventing frailty and depression, [6, 24] which provide more opportunities to go outside [24, 32]. Besides, attending any social activities in itself provides opportunities to walk because attendees have to move to the specific place where the activities take place. On the other hand, only paid work showed a protective relationship with walking time among those who walked less than 60 min/day in 2016. Although earlier studies reported that employment status did not have a significant association with physical activity level [33] while other social participation did, [17, 30] our study among older adults who walk less than 60 min at baseline showed the opposite result. In the literature, commuting to the work place contributes to the maintenance of walking time among office workers considering that they walk from their house to the station, climb stairs, and walk from the station to their office [34, 35]. Thus, we assume that individuals with paid work basically walk longer than do those without paid work. Moreover, when employees quit their job, their walking time will decrease as well. However, the extent of the decrease in walking time when older adults quit their job can be much bigger and more significant compared to when they quit other types of social participation. This aspect warrants further study, because we were unable to examine changes in the status of each type of social participation from 2016 to 2019. Moreover, the frequency of attending paid work was higher than that of attending other types of social participation (see supplemental material 1). This implies that even though paid work does not have a strong association with walking time among those who walk enough, it offers good opportunities to maintain walking time for older adults who do not walk enough otherwise.

The main contribution of our research is that according to our review of the literature, we were the first to conduct a large-scale longitudinal study on types of social participation that affect changes in walking time after 3 years. By stratifying and analyzing based on the average walking time at the baseline, our study verified the relationship between social participation and walking time in older adults who walked less than 60 min and those who walked more than 60 min. The remaining findings imply that connecting older adults with appropriate forms of social participation according to their current walking time is essential for increasing and maintaining time more efficiently. This finding will contribute to the achievement of the goals of the *UN Decade of Healthy Aging 2020–2030*, which suggests improving the proportion of the population that feels safe walking alone in the area in which they live, including older people [36]. To achieve a healthy society with a long lifespan, policymakers should promote an increase the average walking time of older adults, [1, 3] and our study suggests that encouraging social participation helps achieve this goal.

This study has several limitations. First, we used panel data that responded to both 2016 and 2019 (the portion of enrolled local residents analyzed was 36.2% of the baseline survey), and this method can cause selection bias. The respondents who participated in both surveys may have a higher self-management ability and health consciousness than the general population does. Second, the walking time used in the analysis was based on self-reported answers; thus, the average walking time used in this study may differ from that measured with, for example, an accelerometer [37]. Moreover, we were unable to examine the changes in the status of each type of social participation from 2016 to 2019. Therefore, further studies should be conducted to verify whether participation status (e.g., start/continue/quit participating in social activities) exerts different effects on walking time. In addition, the frequency of participation can be another possible independent predictor of the outcome, which requires further study. Furthermore, the forms of social participation may overlap with one another. For example, club activities for senior citizens may include volunteering, walking/physical activity, and cultural activity. However, the manner in which they are organized may be different from those of other forms of social participation. Finally, because the average walking time was converted to “15 min/day,” “45 min/day,” “75 min/day,” or “105 min/day” for analysis, the floor effect cannot be excluded. Therefore, especially in the group for which the walking time was less than 60 min at the baseline, because of the floor effect, the walking time decreases that occur with aging may be smaller than the actual decrease. Thus, in this study, even if the group with social participation of fewer than 60 min had the effect of suppressing the decrease in walking time, the effect could not be sufficiently detected.

## Conclusions

The results of this study suggest that having paid work can help older adults whose walking time is less than 60 min per day to suppress the decline in walking time and that almost all social participation can increase the time of those who walk less than or equal to 60 min per day. Further interventional research to determine the causal relationship between social participation and older adults’ walking time is necessary.

**Table 1:** Characteristics of individuals according to walking time at baseline

Walking time at baseline	< 60 min/day	≥ 60 min/day	*p*
N	34,542	22,500	
Male (%)	16,282 (47.1)	11,189 (49.7)	< 0.001
Age (%)			
65–69 years	11,198 (32.4)	7785 (34.6)	< 0.001
70–74 years	10,117 (29.3)	7029 (31.2)	
75–79 years	8120 (23.5)	5045 (22.4)	
80–84 years	3907 (11.3)	2074 (9.2)	
≥ 85 years	1200 (3.5)	567 (2.5)	
Marital status (%)			
Married	25,726 (74.5)	17,354 (77.1)	< 0.001
Widowed	5853 (16.9)	3342 (14.9)	
Divorced	1514 (4.4)	929 (4.1)	
Never married	1019 (3.0)	640 (2.8)	
Others/Missing	430 (1.2)	235 (1.0)	
Educational attainment (%)			
< 10 years	8360 (24.2)	5675 (25.2)	0.037
10–12 years	15,021 (43.5)	9723 (43.2)	
≥ 13 years	10,829 (31.4)	6897 (30.7)	
Others/Missing	332 (1.0)	205 (0.9)	
Household income (%)			
< 2,000,000 JPY	13,129 (38.0)	7864 (35.0)	< 0.001
2,000,000–3,999,999 JPY	12,055 (34.9)	8391 (37.3)	
≥ 4,000,000 JPY	3526 (10.2)	2466 (11.0)	
Missing	5832 (16.9)	3779 (16.8)	
BMI (%)			
< 18.5	2251 (6.5)	1420 (6.3)	< 0.001
18.5–24.9	24,031 (69.6)	16,566 (73.6)	
≥ 25.0	7736 (22.4)	4227 (18.8)	
Missing	524 (1.5)	287 (1.3)	
IADL (%)			
Dependent	31,591 (91.5)	21,031 (93.5)	< 0.001
Independent	2508 (7.3)	1222 (5.4)	
Missing	443 (1.3)	247 (1.1)	
Drink (%)			
No	19,940 (57.7)	12,393 (55.1)	< 0.001
Yes	14,057 (40.7)	9791 (43.5)	
Missing	545 (1.6)	316 (1.4)	
Smoke (%)			
No	25,194 (72.9)	15,897 (70.7)	< 0.001
Yes	9104 (26.4)	6468 (28.7)	
Missing	244 (0.7)	135 (0.6)	
Volunteer (%)			
No	23,183 (67.1)	14,502 (64.5)	< 0.001
Yes	7675 (22.2)	5598 (24.9)	
Missing	3684 (10.7)	2400 (10.7)	
Sports (%)			
No	18,408 (53.3)	11,064 (49.2)	< 0.001
Yes	11,520 (33.4)	8437 (37.5)	
Missing	4614 (13.4)	2999 (13.3)	
Hobby (%)			
No	15,973 (46.2)	9950 (44.2)	< 0.001
Yes	15,430 (44.7)	10,444 (46.4)	
Missing	3139 (9.1)	2106 (9.4)	
Senior (%)			
No	26,171 (75.8)	16,891 (75.1)	0.151
Yes	4891 (14.2)	3300 (14.7)	
Missing	3480 (10.1)	2309 (10.3)	
Neighborhood (%)			
No	19,537 (56.6)	11,734 (52.2)	< 0.001
Yes	11,706 (33.9)	8573 (38.1)	
Missing	3299 (9.6)	2193 (9.7)	
Learning (%)			
No	25,499 (73.8)	16,337 (72.6)	0.005
Yes	5478 (15.9)	3760 (16.7)	
Missing	3565 (10.3)	2403 (10.7)	
Health (%)			
No	26,566 (76.9)	16,744 (74.4)	< 0.001
Yes	4618 (13.4)	3519 (15.6)	
Missing	3358 (9.7)	2237 (9.9)	
Skills (%)			
No	27,281 (79.0)	17,151 (76.2)	< 0.001
Yes	4283 (12.4)	3314 (14.7)	
Missing	2978 (8.6)	2035 (9.0)	
Paid work (%)			
No	22,566 (65.3)	12,206 (54.2)	< 0.001
Yes	9485 (27.5)	8715 (38.7)	
Missing	2491 (7.2)	1579 (7.0)	

Independent variables: nine types of social participation, volunteer groups (volunteer), sports groups or clubs (sports), hobby groups (hobby), senior citizen clubs (senior), neighborhood associations (neighborhood), learning or cultural groups (learning), long term care prevention or health promoting activity groups (health), activities to teach skills or pass on experiences to others (skills), paid work (work). A chi-square test and t test were conducted to examine the difference of each proportion

**Table 2:** Multiple regression analysis of differences of walking time from 2016 to 2019 in older adults who walked < 60 min/day at baseline

*N* = 34,542	**Crude Model**				**Model 1**				**Model 2**			
	**β**	**t**	**95% CI**	*p*	**β**	**t**	**95% CI**	*p*	**β**	**t**	**95% CI**	*p*
Volunteer	− 0.12	− 0.38	(− 0.75 to 0.51)	0.703	− 0.07	− 0.23	(− 0.70 to 0.55)	0.816	− 0.01	− 0.04	(− 0.64 to 0.62)	0.969
Sports	0.25	0.88	(− 0.31 to 0.82)	0.379	0.20	0.70	(− 0.37 to 0.77)	0.487	0.30	1.03	(− 0.27 to 0.88)	0.301
Hobby	− 0.59	− 2.15	(− 1.13 to − 0.05)	0.031	− 0.57	− 2.05	(− 1.11 to − 0.03)	0.040	− 0.48	− 1.70	(− 1.03 to 0.07)	0.090
Senior	− 0.31	− 0.82	(− 1.06 to 0.43)	0.412	0.47	1.22	(− 0.29 to 1.23)	0.224	0.41	1.05	(− 0.36 to 1.17)	0.296
Neighborhood	− 0.28	− 0.99	(− 0.84 to 0.28)	0.323	− 0.23	− 0.82	(− 0.79 to 0.32)	0.411	− 0.22	− 0.78	(− 0.78 to 0.34)	0.436
Learning	− 0.07	− 0.20	(− 0.79 to 0.64)	0.839	− 0.08	− 0.23	(− 0.80 to 0.63)	0.820	0.09	0.24	(− 0.64 to 0.82)	0.811
Health	0.30	0.78	(− 0.46 to 1.06)	0.438	0.47	1.21	(− 0.30 to 1.24)	0.227	0.48	1.23	(− 0.29 to 1.25)	0.219
Skills	0.25	0.62	(− 0.54 to 1.03)	0.534	0.29	0.71	(− 0.50 to 1.07)	0.475	0.42	1.05	(− 0.37 to 1.21)	0.294
Paid Work	3.40	11.44	(2.82 to 3.99)	< 0.001	3.01	9.76	(2.41 to 3.62)	< 0.001	3.02	9.68	(2.41 to 3.63)	< 0.001

Multiple regression analysis was conducted to compare those who have each type of social participation to those who do not (control group

Crude Model: Each status of nine types of social participation was included. Model 1: Crude + sex and age

Model 2: Model 1 + marital status, educational attainment, household income, BMI, IADL, drink and smoke

Independent variables: nine types of social participation: volunteer groups (volunteer), sports groups or clubs (sports), hobby groups (hobby), senior citizen clubs (senior), neighborhood associations (neighborhood), learning or cultural groups (learning), long-term care prevention or health-promoting activity groups (health), activities to teach skills or pass on experiences to others (skills), and paid work (work)

**Table 3:** Multiple regression analysis of differences of walking time from 2016 to 2019 in older adults who walked ≥ 60 min/day at baseline

*N* = 22,500	**Crude Model**				**Model 1**				**Model 2**			
	**β**	**t**	**95% CI**	***p***	**β**	**t**	**95% CI**	**p**	**β**	**t**	**95% CI**	***p***
Volunteer	2.19	4.72	(1.28 to 3.10)	< 0.001	2.34	5.04	(1.43 to 3.25)	< 0.001	2.15	4.61	(1.23 to 3.06)	< 0.001
Sports	3.24	7.61	(2.41 to 4.07)	< 0.001	3.33	7.81	(2.49 to 4.16)	< 0.001	2.88	6.68	(2.04 to 3.73)	< 0.001
Hobby	1.89	4.59	(1.08 to 2.70)	< 0.001	2.19	5.26	(1.37 to 3.00)	< 0.001	1.71	4.07	(0.89 to 2.54)	< 0.001
Senior	− 0.07	− 0.13	(− 1.17 to 1.03)	0.900	1.00	1.73	(− 0.13 to 2.12)	0.083	1.27	2.20	(0.14 to 2.40)	0.028
Neighborhood	1.75	4.18	(0.93 to 2.57)	< 0.001	1.77	4.23	(0.95 to 2.59)	< 0.001	1.70	4.04	(0.87 to 2.52)	< 0.001
Learning	1.87	3.50	(0.82 to 2.91)	< 0.001	2.31	4.30	(1.26 to 3.37)	< 0.001	1.65	3.03	(0.58 to 2.72)	0.002
Health	1.20	2.20	(0.13 to 2.28)	0.028	1.90	3.43	(0.81 to 2.98)	< 0.001	1.74	3.14	(0.65 to 2.82)	0.002
Skills	2.18	3.90	(1.09 to 3.28)	< 0.001	2.32	4.15	(1.22 to 3.41)	< 0.001	1.95	3.46	(0.85 to 3.05)	< 0.001
Paid Work	1.48	3.58	(0.67 to 2.29)	< 0.001	0.61	1.43	(− 0.23 to 1.46)	0.152	0.64	1.49	(− 0.20 to 1.49)	0.136

Multiple regression analysis was conducted to compare those who have each type of social participation to those who do not (control group)

Crude Model: Each status of nine types of social participation was included. Model 1: Crude + sex and age

Model 2: Model 1 + marital status, educational attainment, household income, BMI, IADL, drink and smoke

Independent variables: nine types of social participation: volunteer groups (volunteer), sports groups or clubs (sports), hobby groups (hobby), senior citizen clubs (senior), neighborhood associations (neighborhood), learning or cultural groups (learning), long-term care prevention or health-promoting activity groups (health), activities to teach skills or pass on experiences to others (skills), and paid work (work)

## Supplementary Information

**Additional file 1.**

## Data Availability

Data were derived from the JAGES. All enquiries are to be addressed at the data management committee via e-mail: dataadmin.ml@jages.net. All datasets from JAGES hold ethical or legal restrictions for public deposition due to the inclusion of sensitive information from the participants. Following the regulation of the local governments, which cooperated with the survey, the JAGES data management committee has imposed such restrictions upon the data.
