# Supplementary material for: Social participation and change in walking time among older adults: a 3-year longitudinal study from the JAGES
Source: BMC Geriatr. 2022 Mar 22;22:238. doi: 10.1186/s12877-022-02874-2 (PMC8941795; doi:10.1186/s12877-022-02874-2)
Supplement: Supplementary file 1 — Additional file 1. [file 12877_2022_2874_MOESM1_ESM.docx]

Supplemental material 1 Frequency of social participation

| Walking time at baseline | <60 min/day | ≥60 min/day | p |
| --- | --- | --- | --- |
| N | 34542 | 22500 |  |
| Volunteer(%) |  |  | <0.001 |
| Four or more times per week | 425 (1.2) | 420 (1.9) |  |
| Two or three times per week | 1040 (3.0) | 715 (3.2) |  |
| Once per week | 1059 (3.1) | 687 (3.1) |  |
| Once to three times per month | 2711 (7.8) | 1878 (8.3) |  |
| A few times per year | 2440 (7.1) | 1898 (8.4) |  |
| No participation | 23183 (67.1) | 14502 (64.5) |  |
| Missing | 3684 (10.7) | 2400 (10.7) |  |
| Sports (%) |  |  | <0.001 |
| Four or more times per week | 1598 (4.6) | 1857 (8.3) |  |
| Two or three times per week | 3514 (10.2) | 2362 (10.5) |  |
| Once per week | 2658 (7.7) | 1597 (7.1) |  |
| Once to three times per month | 2385 (6.9) | 1614 (7.2) |  |
| A few times per year | 1365 (4.0) | 1007 (4.5) |  |
| No participation | 18408 (53.3) | 11064 (49.2) |  |
| Missing | 4614 (13.4) | 2999 (13.3) |  |
| Hobby(%) |  |  | <0.001 |
| Four or more times per week | 1327 (3.8) | 1395 (6.2) |  |
| Two or three times per week | 3530 (10.2) | 2308 (10.3) |  |
| Once per week | 3208 (9.3) | 2000 ( 8.9) |  |
| Once to three times per month | 5318 (15.4) | 3276 (14.6) |  |
| A few times per year | 2047 (5.9) | 1465 ( 6.5) |  |
| No participation | 15973 (46.2) | 9950 (44.2) |  |
| Missing | 3139 (9.1) | 2106 (9.4) |  |
| Senior (%) |  |  | <0.001 |
| Four or more times per week | 134 (0.4) | 147 (0.7) |  |
| Two or three times per week | 458 (1.3) | 333 (1.5) |  |
| Once per week | 396 (1.1) | 280 (1.2) |  |
| Once to three times per month | 1500 (4.3) | 991 (4.4) |  |
| A few times per year | 2403 (7.0) | 1549 (6.9) |  |
| No participation | 26171 (75.8) | 16891 (75.1) |  |
| Missing | 3480 (10.1) | 2309 (10.3) |  |
| Neighborhood (%) |  |  | <0.001 |
| Four or more times per week | 133 (0.4) | 126 (0.6) |  |
| Two or three times per week | 299 (0.9) | 226 (1.0) |  |
| Once per week | 387 (1.1) | 275 (1.2) |  |
| Once to three times per month | 2553 (7.4) | 1859 (8.3) |  |
| A few times per year | 8334 (24.1) | 6087 (27.1) |  |
| No participation | 19537 (56.6) | 11734 (52.2) |  |
| Missing | 3299 (9.6) | 2193 (9.7) |  |
| Learning (%) |  |  | <0.001 |
| Four or more times per week | 143 (0.4) | 131 (0.6) |  |
| Two or three times per week | 463 (1.3) | 344 (1.5) |  |
| Once per week | 913 (2.6) | 588 (2.6) |  |
| Once to three times per month | 2227 (6.4) | 1397 (6.2) |  |
| A few times per year | 1732 (5.0) | 1300 (5.8) |  |
| No participation | 25499 (73.8) | 16337 (72.6) |  |
| Missing | 3565 (10.3) | 2403 (10.7) |  |
| Health (%) |  |  | <0.001 |
| Four or more times per week | 290 (0.8) | 379 (1.7) |  |
| Two or three times per week | 551 (1.6) | 443 (2.0) |  |
| Once per week | 641 (1.9) | 426 (1.9) |  |
| Once to three times per month | 1049 (3.0) | 741 (3.3) |  |
| A few times per year | 2087 (6.0) | 1530 (6.8) |  |
| No participation | 26566 (76.9) | 16744 (74.4) |  |
| Missing | 3358 (9.7) | 2237 (9.9) |  |
| Skills (%) |  |  | <0.001 |
| Four or more times per week | 301 (0.9) | 344 (1.5) |  |
| Two or three times per week | 523 (1.5) | 405 (1.8) |  |
| Once per week | 536 (1.6) | 418 (1.9) |  |
| Once to three times per month | 1156 (3.3) | 804 ( 3.6) |  |
| A few times per year | 1767 (5.1) | 1343 (6.0) |  |
| No participation | 27281 (79.0) | 17151 (76.2) |  |
| Missing | 2978 (8.6) | 2035 (9.0) |  |
| Paid Work (%) |  |  | <0.001 |
| Four or more times per week | 4553 (13.2) | 5044 (22.4) |  |
| Two or three times per week | 2280 (6.6) | 1927 (8.6) |  |
| Once per week | 637 (1.8) | 359 (1.6) |  |
| Once to three times per month | 970 (2.8) | 648 (2.9) |  |
| A few times per year | 1045 (3.0) | 737 (3.3) |  |
| No participation | 22566 (65.3) | 12206 (54.2) |  |
| Missing | 2491 (7.2) | 1579 (7.0) |  |

Chi-square tests were conducted to examine the distribution of frequency of each social participation

Supplemental material 2. Adjusted R square of each multiple linear regression

|  | <60 group  model1 | <60 group  model2 | ≥60 group  model1 | ≥60 group  model2 |
| --- | --- | --- | --- | --- |
| Volunteer | 0.0032 | 0.0041 | 0.0050 | 0.0088 |
| Sports | 0.0032 | 0.0041 | 0.0067 | 0.0099 |
| Hobby | 0.0033 | 0.0042 | 0.0052 | 0.0086 |
| Senior | 0.0033 | 0.0042 | 0.0038 | 0.0079 |
| Neighborhood | 0.0032 | 0.0042 | 0.0045 | 0.0084 |
| Learning | 0.0032 | 0.0042 | 0.0045 | 0.0081 |
| Health | 0.0032 | 0.0042 | 0.0043 | 0.0082 |
| Skills | 0.0032 | 0.0041 | 0.0045 | 0.0083 |
| Paid work | 0.0059 | 0.0068 | 0.0038 | 0.0078 |
